# Supplementary material for: Extracellular Vesicles-Induced Cell Homing and Odontogenesis via microRNA Signaling for Dentin Regeneration
Source: Int J Mol Sci. 2025 Jul 25;26(15):7182. doi: 10.3390/ijms26157182 (PMC12346742; doi:10.3390/ijms26157182)
Supplement: Supplementary file 1 [file ijms-26-07182-s001.zip › ijms-3765355-supplementary.pdf]

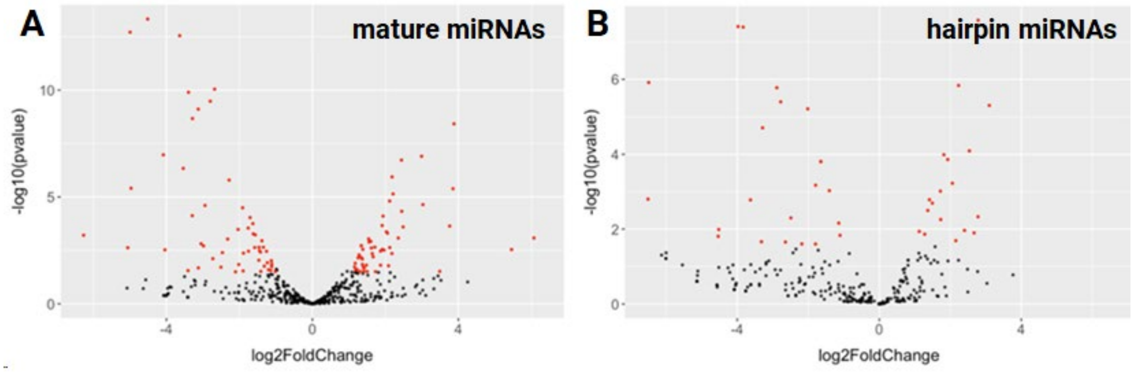

Figure S1. Volcano plots of mature (A) and hairpin (B) micro ribonucleic acids (miRNAs): EV-G versus EV-O. Extracellular vesicles (EVs) were prepared from conditioned media under regular growth medium (EV-G) or odontogenic differentiation medium (EV-O).

Table S1. List of significantly up/down-expressed hairpin micro ribonucleic acids (miRNAs): Exo-O versus Exo-G in over 0.01% population. Extracellular vesicles (EVs) were prepared from conditioned media of rabbit (Oryctolagus cuniculus: ocu) dental pulp stem cells under regular growth medium (EV-G) or odontogenic differentiation medium (EV-O).

miRNA expression: EV-O > EV-G	log2(fold change)	p-value	miRNA expression: EV-O < EV-G	log2(fold change)	p-value
**ocu-miR-708**	6.48	1.21E-06	**ocu-miR-146a**	-3.10	4.98E-06
**ocu-miR-509c**	6.46	0.00158	**ocu-miR-503**	-2.79	2.63E-08
**ocu-miR-498**	4.60	0.01559	**ocu-miR-122**	-2.55	8.14E-05
**ocu-miR-2387**	3.99	3.87E-08	**ocu-miR-155**	-2.24	1.46E-06
**ocu-miR-182**	3.84	4.01E-08	**ocu-miR-186**	-2.09	0.00060
**ocu-miR-328**	3.63	0.00166	**ocu-miR-183**	-2.09	0.02036
**ocu-miR-520e**	3.31	0.02169	**ocu-miR-30a**	-1.94	0.00014
**ocu-miR-134**	3.27	1.98E-05	**ocu-let-7f-1**	-1.82	0.00010
**ocu-miR-29b-2**	2.88	1.67E-06	**ocu-miR-181b-2**	-1.75	0.00558
**ocu-miR-29b-1**	2.77	3.98E-06	**ocu-miR-93**	-1.72	0.00097
**ocu-miR-512a**	2.62	0.02213	**ocu-miR-425**	-1.50	0.00203
**ocu-miR-371**	2.50	0.00502	**ocu-miR-142**	-1.41	0.00164
**ocu-miR-744**	2.19	0.02484	**ocu-let-7f-2**	-1.36	0.00318
**ocu-miR-542**	2.01	6.13E-06	**ocu-miR-151**	-1.28	0.01366
**ocu-miR-140**	1.79	0.00067	**ocu-miR-451**	-1.13	0.01154
**ocu-miR-12090**	1.78	0.02501			
**ocu-miR-101-2**	1.65	0.00016			
**ocu-miR-101-1**	1.65	0.00018			
**ocu-miR-214**	1.41	0.00094			
**ocu-miR-23b**	1.14	0.00693			
**ocu-miR-29a**	1.10	0.01467			

Table S2. List of significantly up/down-expressed both mature and hairpin micro ribonucleic acids (miRNAs): Exo-O versus Exo-G in over 0.01% population. Extracellular vesicles (EVs) were prepared from conditioned media of rabbit (Oryctolagus cuniculus: ocu) dental pulp stem cells under regular growth medium (EV-G) or odontogenic differentiation medium (EV-O).

miRNA expression: EV-O > EV-G	miRNA expression: EV-O < EV-G
**ocu-miR-708**	**ocu-miR-146a**
**ocu-miR-328**	**ocu-miR-503**
**ocu-miR-29b**	**ocu-miR-122**
**ocu-miR-744**	**ocu-let-7f**
**ocu-miR-542**	**ocu-miR-151**
**ocu-miR-12090**	
**ocu-miR-101**	
**ocu-miR-214**	
**ocu-miR-29a**	
